# Targeted imaging of esophageal adenocarcinoma with a near-infrared fluorescent peptide

**DOI:** 10.1186/s12876-021-01840-3

**Published:** 2021-06-12

**Authors:** Xiaoyu Kang, Meng Li, Lei liu, Shaopeng Liu, Hao Hu, Rui Zhang, Siming Ning, Zuhong Tian, Yanglin Pan, Xuegang Guo, Kaichun Wu

**Affiliations:** 1 State Key Laboratory of Cancer Biology, National Clinical Research Center for Digestive Diseases and Xijing Hospital of Digestive Diseases, Fourth Military Medical University, 127 Changle West Road, Xi’an, 710032 Shaanxi People’s Republic of China; 2 Biotechnology Center, School of Pharmacy, Fourth Military Medical University, Xi’an, Shaanxi People’s Republic of China; 3 Department of Gastroenterology, Tangdu Hospital of the Fourth Military Medical University, Xi’an, Shaanxi People’s Republic of China; 4 Department of Critical Care Medicine, Shaanxi Provincial Cancer Hospital, Xi’an, Shaanxi People’s Republic of China; 5 College of Medicine, Xi’an Jiaotong University, Xi’an, Shaanxi People’s Republic of China

**Keywords:** Esophageal carcinoma, Peptide, Near infrared, Probe

## Abstract

**Background:**

Targeted optical imaging offers a noninvasive and accurate method for the early detection of gastrointestinal tumors, especially for flat appearances. In our previous study, a sequence of SNFYMPL (SNF) was identified as a specific peptide to bind to esophageal carcinoma using phage-display technology. This study aimed to evaluate the tumor-targeting efficacy of Cy5.5-conjugated SNF probe for imaging of esophageal carcinoma in vitro and in vivo.

**Methods:**

The SNF-Cy5.5 probe was synthesized and then identified using High Performance Liquid Chromatography (HPLC) and mass spectrometry (MS). Confocal fluorescence imaging and Flow cytometry analysis were performed to evaluate the binding specificity and the receptor binding affinity of SNF-Cy5.5 to OE33. In vivo imaging was performed to evaluate the targeting ability of SNF-Cy5.5 to esophageal carcinoma.

**Results:**

The confocal imaging and flow cytometry analysis showed that SNF-Cy5.5 bound specifically to the plasma membrane of OE33 cells with a high affinity. In vivo, for non-block group, SNF-Cy5.5 probe exhibited rapid OE33 tumor targeting during 24 h p.i. and excellent tumor-to-background contrast at 2 h p.i. For the block group, SNF-Cy5.5 was not observed in the mice after 4 h p.i. Ex vivo imaging also revealed that a higher fluorescent signal intensity value of the tumors was clearly observed in the non-block group than that in the block group (2.6 ± 0.32 × 10^9^ vs. 0.8 ± 0.08 × 10^9^, p < 0.05).

**Conclusions:**

SNF-Cy5.5 was synthesized and characterized with a high efficiency and purity. The higher affinity, specificity, and tumor targeting efficacy of SNF-Cy5.5 were confirmed by in vitro and in vivo tests. SNF-Cy5.5 is a promising optical probe for the imaging of esophageal adenocarcinoma.

**Supplementary Information:**

The online version contains supplementary material available at 10.1186/s12876-021-01840-3.

## Background

A rapidly increasing trend has presented with regard to the incidence and mortality of esophageal adenocarcinoma (EAC) across the globe for decades, and due to westernized lifestyle, Some developing countries, such as China, embrace same challenge as well [1, 2]. Early diagnosis and treatment of EAC is associated with improved patients’ survival. Opportunistic biopsy using White-light endoscopy has proven to have limited efficacy for diagnosing EAC in routine. In addition, the optical white imaging is prone to receive false signals from self-absorption and scattering of humans’ tissues. Another limitation is the fact that flat lesions are hardly detected due to non-targeted imaging.

Absorption and auto-flourescence of biological tissues is low in the near-infrared (NIR) spectrum (650–900 nm) [3]. Previous studies have revealed that NIR fluorescence (NIRF) imaging has been used to visualize or measure various biological closely connected with diseases’ process in the cellular or molecular level [4–8], which provided more information for us to apply this promising technique to early detection of neoplasm originated from hollow organs. A NIR imaging probe is essential for NIR fluorescent imaging. This probe typically comprised of both targeting moiety and NIR dyes (eg. Cy5.5) The targeting moieties, including nanoparticles, small molecules, peptides, proteins and antibodies or its fragments, could specifically interact with biomarkers in the process of derivation and evolution of tumors. Compared with other moieties, short peptides had the following advantages: easier to be synthesized and modified; rapider blood clearance; increased permeability; lower toxicity and lower immunogenicity [9, 10].

In a previous study, we identified a seven amino acids—peptide sequence, which could specifically bind to human esophageal dysplasia or adenocarcinoma [11]. In our study, we conjugated peptide SNFYMPL with NIRF dye Cy5.5 through the linker (GGGSK) and we evaluated the tumor-targeting efficacy of the probe in vitro and in vivo.

## Methods

### Synthesis of SNF-Cy5.5

To maintain the original structure of peptide SNFYMPL and productive conjugation with Cy5.5-NHS, a GGGSK linker was added to the C-terminus of SNFYMPL. The peptide-linker SNFYMPLGGGSK were synthesized through using standard solid-phase fluorenylmethoxycarbonyl chemistry as previously reported [12]. Cy5.5 monofunctional NHS ester was purchased from GE Healthcare (Piscataway, NJ, USA).

Cy5.5 monofunctional NHS ester (0.50 mg, 0.443 μmol. 1 equiv.) was dissolved in 200 μl of Dimethyl sulfoxide (DMSO)and mixed with SNF peptide (0.54 mg, 0.443 μmol, 1 equiv) in the dark. Then triethylamine (5 µl) was added to the mixed solution. The reaction mixture was stirred overnight in dark at room temperature. After removal of the solvent, The crude product was dissolved in a solution of 1:1 acetonitrile and H_2_O and purified by HPLC with a C18 column (25 cm × 10 mm) using a gradient mobile phase beginning from 5% solvent A [0.1% trifluoroacetic acid(TFA) in acetonitrile] and 95%solvent B (0.1% TFA) in water) to 70% solvent A and 30%solvent B at 30 min. Finally, the coloured peak was collected and lyophilized. The ultimate purity of the peptides was confirmed with an analytical C18-column. SNF-Cy5.5 was also analyzed by mass spectrometry.

### Cell culture and condition

The human-derived esophageal adenocarcinoma cell line OE33 and immortalized epithelial cell line Het-1A were purchased from ATCC (Manassas, VA). OE33 cells were cultured in RPMI 1640 (Gibco) containing 10% FBS (Gibco), and 100 U/mL streptomycin/penicillin. Het-1A cells were cultured in Bronchial Epithelial Cell Growth Medium (BEBM), along with the additives obtained from Lonza/Clonetics Corporation as a kit (Catalog No.CC-3170). All cells were incubated at 37 °C in a 5% CO_2_ humidified atmosphere.

### Fluorescence imaging

OE33 and Het-1A cells were grown in 4-chamber slides for 24 h, then the cells were washed 3 times in PBS and fixed with acetone on ice for 15 min. Blocking was performed by adding 10% normal rabbit serum. The cells were then incubated with 10 μmol/L of the SNF-Cy5.5 at dark. In the block group, the cells were incubated with 10 μmol/l of SNF-Cy5.5 and 50 μmol/l of unlabeled SNF peptide. The cells were washed with PBS for 3 times after fluorescently-labeled peptide. 4',6-diamidino-2-phenylindole (DAPI) (Invitrogen, CA, USA) was dropped onto the cell chambers and incubated with cells for 15 min. After a drop of Prolong Gold anti-fade reagent was added onto the slides, the images were acquired using a FLUOVIEW FV1000 laser scanning confocal microscope (Olympus, Tokyo, Japan).

### Flow cytometry analysis

We also performed flow cytometry to validate Preferential binding of the SNF-Cy5.5 to OE33 cells. Cell triplicates counting about 10^6^ were prepared. OE33 cells were the cells were trypsinized, centrifuged at 800 rpm for 5 min, then collected and washed with ice-cold PBS thrice. The cells were incubated with SNF-Cy5.5, SNF-Cy5.5 with block peptide and PBS at the concentration as previously stated for 30 min on ice. These cells were then washed by ice-cold PBS thrice to clean unspecific binding. Finally, fluorescence-activated cell sorting (FACS) was used to record and analyze the results.

### Animal model

Athymic female nude male BALB/c mice (4–6 weeks, 20–25 g) were obtained from the Laboratory Animal Center of Fourth Military Medical University (FMMU), with the care and treatment of these animals performed in accordance with FMMU animal protocols. When female athymic nude mice grew until about 4–6 weeks old, weighing about 20–25 g, about 5 × 10^6^ OE33 cells suspended in 200 μL of serum-free RPMI 1640 were injected into the right shoulder of mice. The cells were allowed to grow 2 weeks until tumors’ size was was 5–10 mm. The animal studies were registered and approved by the Animal Welfare and Ethics Committee of Fourth Military Medical School.

### Targeting ability of SNF-Cy5.5 in vivo

In vivo imaging system (IVIS) Imaging System (IVIS Kinetics, Caliper Life Sciences, Hopkinton, MA, USA), was used to assess the tumor targeting efficacy of the SNF-Cy5.5. A Cy5.5 fiter set and Identical illumination settings (eg. lamp voltage, fiters, f/stop, field of views, binning) were used to acquire all images. The mice in the experimental group (n = 3) received a 100 μM of SNF-Cy5.5 tail-intravenously and then exposed to optical imaging of IVIS at various period points after injection. The mice from the block group (n = 3) received a 100 μM of SNF-Cy5.5 mixed with unlabeled SNF peptide (1000 μM) as previously described [5]. After IRI fluorescent imaging of the tumor-bearing mice, the mice were euthanized using 100% CO_2_ in the chamber. Various organs, such as tumor, heart and liver, were excised and subjected to ex vivo fluorescence imaging. The bioluminescent intensities of the regions of interest (ROIs) were measured by Living Image version 4.2 Software. Fluorescent intensity was reported as photons per second per centimeter squared per steradian (p/s/cm^2^/sr).

### Data analysis

All the data are described as means ± standard deviation (SD). Statistical analysis was performed using a Student’s *t* test. To determine tumor contrast, mean fluorescence intensities of the tumor (T) area at the right shoulder of the animal and of the normal tissue (N) at the surrounding tissue were calculated using the ROI function Dividing T by N yielded the contrast between tumor and normal tissue. Statistical analysis of the data was performed using SPSS 23.0 software (Chicago, IL, USA). If p-value < 0.05, Statistical significance was defined.

## Results

### Synthesis of SNF-Cy5.5

**Fig. 1:**
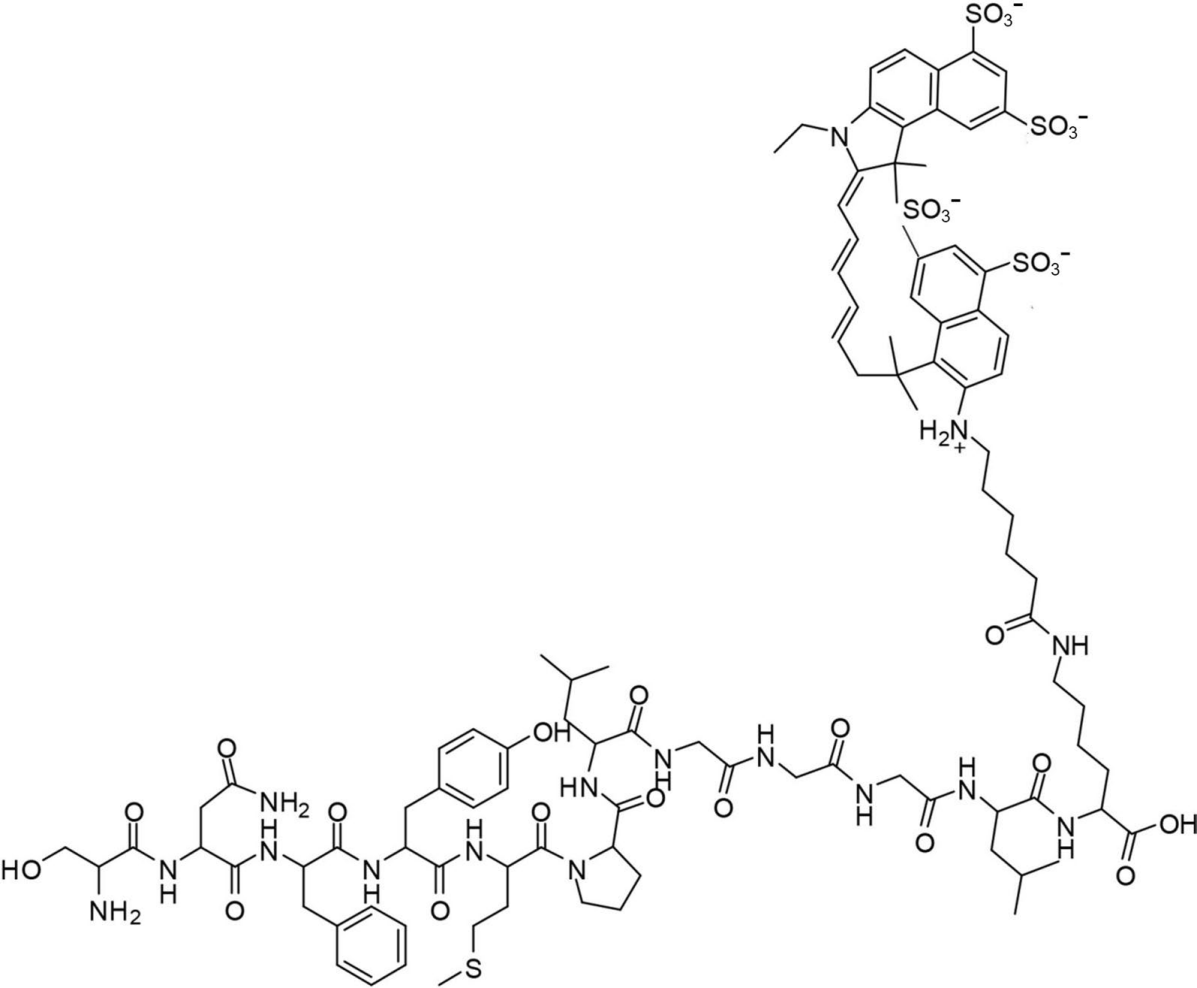
Schematic structure of the SNF-Cy5.5 conjugate

The chemical molecular structure of SNF-Cy5.5 is presented in Fig. 1. The SNF-Cy5.5 peptide probe was isolated through HPLC purification. As shown in the supplemental Fig. 1, the purity of the main product was reported as > 95%. The retention time of SNF-Cy5.5 on HPLC was 10.9 min. High-resolution mass spectrometry confirmed mass-to-charge ratio (m/z) = 2154.18 for SNF-Cy5.5.

### FACS and fluorescence imaging

**Fig. 2:**
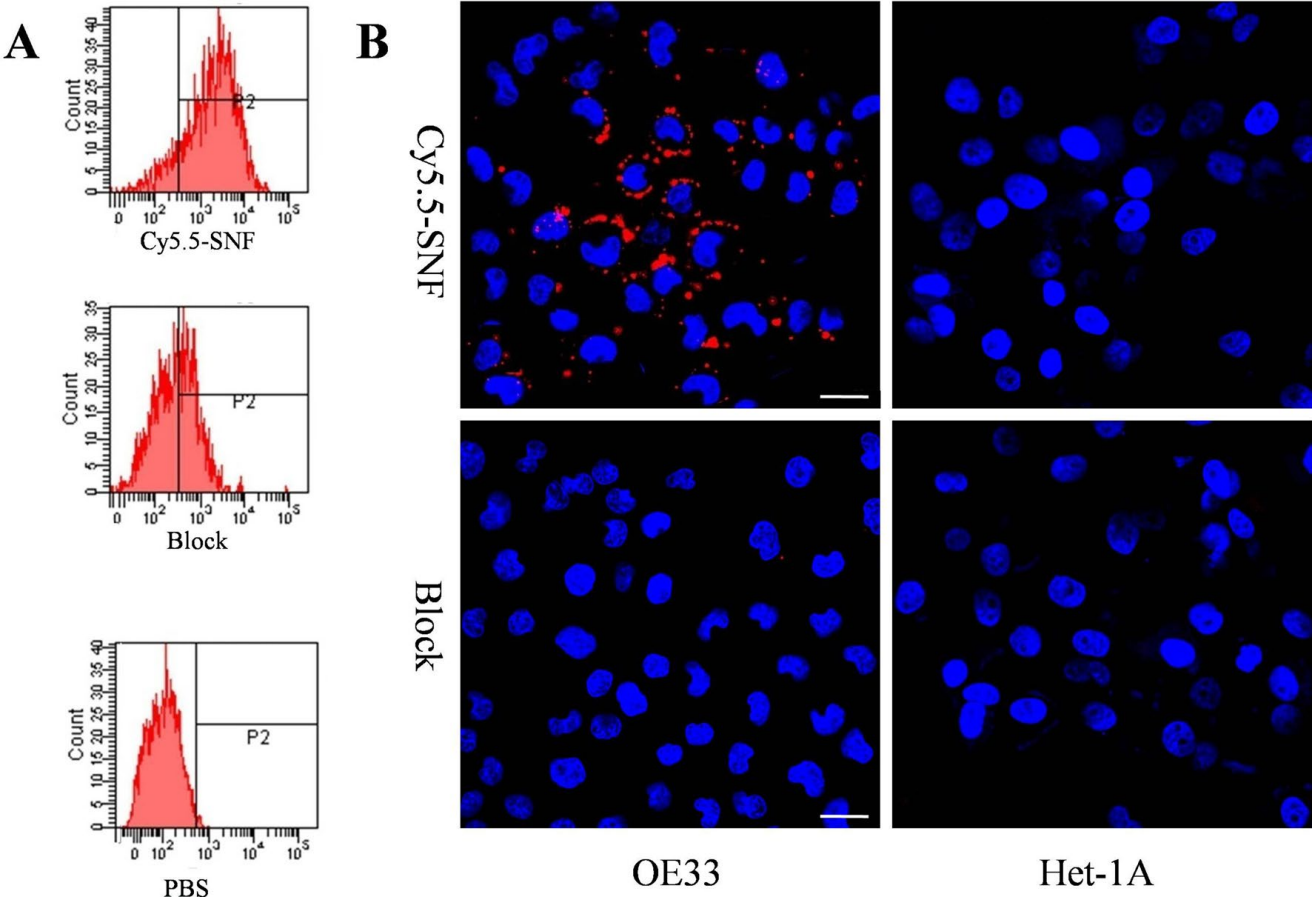
FACS analysis and confocal staining. FACS analysis of OE33 cells incubated with SNF-Cy5.5, SNF-Cy5.5 + unlabeled SNF and PBS (**A**). confocal immunofluorescence of SNF-Cy5.5 location (× 400). Cells were stained with DAPI (nuclear staining) colored in green, and Cy5.5-GX1 colored in red (**B**). The SNF-Cy5.5 binds to the plasma membrane of OE33 cells but little binding to that of the control cells and Het-1A or that of OE33 blocked by unlabeled SNF

After analysis of FACS results, we found that SNF-Cy5.5 had a stronger binding to OE33 cells (Fig. 2A), Fig. 2B showed that the red fluorescence labeled SNF-Cy5.5 was bound to the cytoplasm of OE33 and not to Het-1a cells. By comparison, in the block group, the red fluorescence is hardly observed for OE33 and Het-1a.

### In vivo fluorescence imaging

**Fig. 3:**
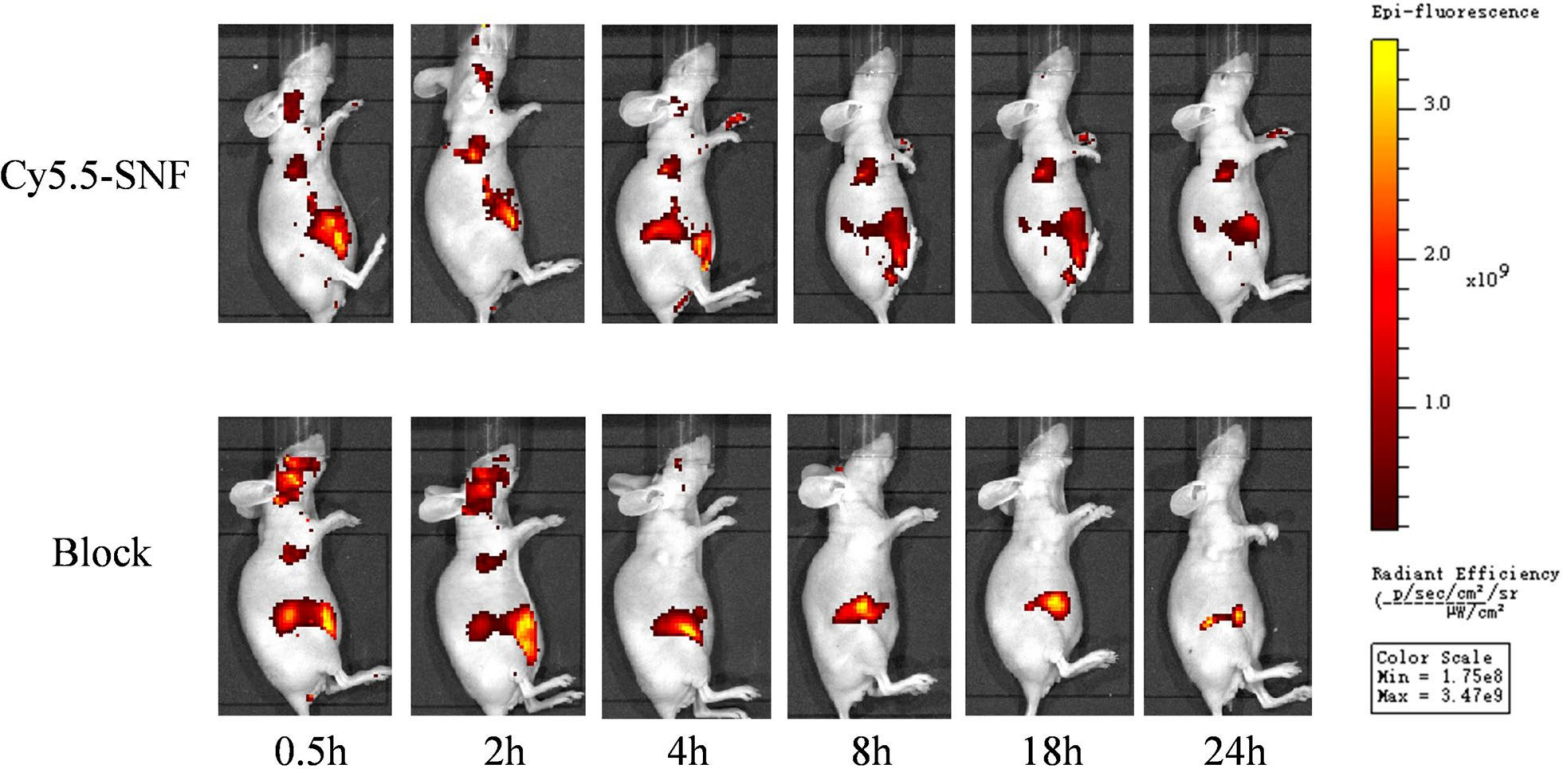
In-vivo imaging. The imaging of fluorescent whole-body is obtained during 24 h post-injection in mice injected with Cy5.5 or SNF-Cy5.5 + unlabeled SNF

To evaluate the targeting efficacy of SNF-Cy5.5 in vivo, the small animal imaging system was used for imaging of OE-33 tumor-bearing nude mice with SNF-Cy5.5 injection (n = 3) or peptide with block (n = 3). The static images were acquired at 0.5, 2, 4, 8, 18, and 24 h p.i. in nude mice models (Fig. 3). The SNF-Cy5.5 probe began to present a high tumor-to-background contrast at 0.5 h p.i. and an excellent contrast at 2 h p.i., but the fluorescence intensity of tumor-to-background contrast decreased after 4 h p.i. In the block group, the fluorescence signal could be found at 0.5 h and 2 h p.i. but not observed in the mice after 4hp.i. Unlabeled SNF peptide significantly reduced OE33 tumor uptake of Cy5.5 SNF compared with the non-block group. The greatest intensity of fluorescence was observed in the hepatic regions from 0.5 to 24 h.

**Fig. 4:**
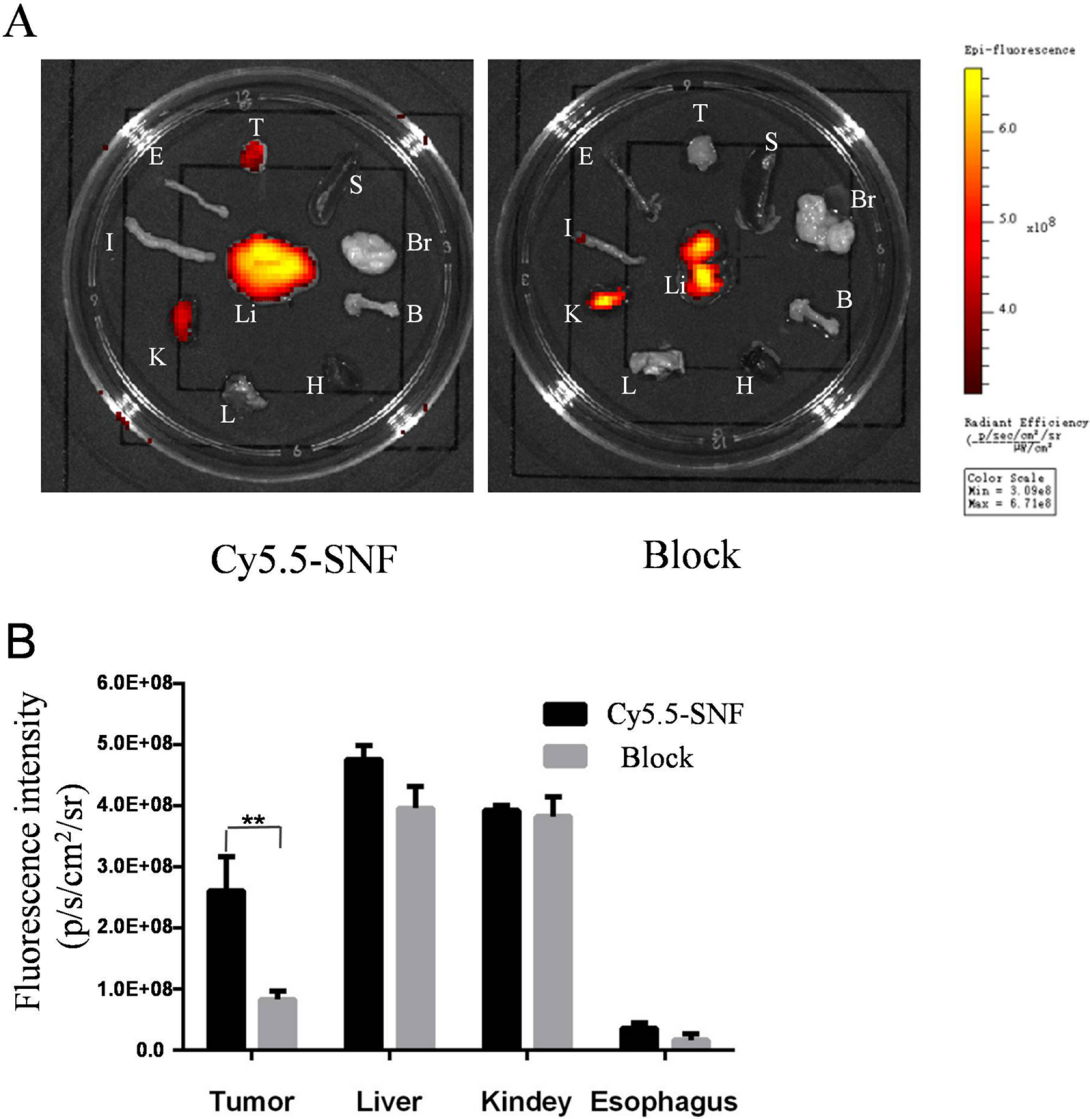
Fluorescent imaging of the desected organs at 24 h post-injection. T: tumor S: spleen Br: brain B: bone H: heart Li: liver L: lung K: kidney I: intestinal E: esophagus (**A**). Quantification analysis of the fluorescent signals of tumor, liver, kidney and esophagus at 24 h post-injection. n = 3. **, p < 0.05 (**B**)

Further evaluation was performed through investigating ex vivo excised organs at 24 h p.i. Figure 4 showed quantified fluorescence intensities of tumor and organs by using ROIs. In the SNF-Cy5.5 group, tumor, liver and kidney had excellent fluorescence intensity compared with that of other organs (Fig. 4A). In the block group, liver and kidney still showed excellent intensity, while the tumor didn’t show it (Fig. 4A). Moreover, the fluorescence intensities of each excised organ were quantified (Fig. 4B). A higher fluorescent signal intensity value of the tumors was clearly observed in the non-block group than that in the block group (2.6 ± 0.32 × 10^9^ vs. 0.8 ± 0.08 × 10^9^, p < 0.05). No significant differences were found with regard to the fluorescence intensities of liver, kidney and esophagus.

## Discussion

Based on advances in molecular biology and high-tech imaging instruments, molecular imaging has provided more opportunities for noninvasive visualization of abnormal molecules events in living subjects. Molecular imaging has been widely applied in unclear medicine. With the aids of positron emission tomography (PET) and single photon emission computed tomography (SPECT), Molecular Imaging and Precision Medicine has been achieved for the diagnosis of different kinds of tumors [13, 14]. In the recent years, efforts are ongoing to apply optical imaging techniques to molecular imaging in vivo [15–17].Optical imaging has several advantages over nuclear imaging in the following aspects: reduced side effects, limited facility requirements, lower financial cost and so on [18]. Therefore, the optical imaging has been a major research field in molecular imaging.

Cy5.5 conjugated peptide SNFYMPL is such a biologically active probe in the current study. The linear-chain sequence SNFYMPL was identified through using the technique of phage-display and results showed a high binding affinity to human esophageal adenocarcinoma cells [11], the ELISA and fluorescence staining studies indicate that SNF could be used as a novel marker for human esophageal adenocarcinoma cells. In our previous research, fluorescein isothiocyanate (FITC) conjugated SNF was applied for the early detection of high-grade barrett’s esophagus and esophageal adenocarcinoma. However, the use of FITC conjugated peptide was only confined to evaluate alterations that arise from the epithelial layer of the tissues, which may leads to the missed diagnose of some flat or invisible lesions [19]. Longer wavelengths, such as NIR would overcome the shortcomings of FITC. Therefore, we synthesized a Cy5.5 conjugated SNF peptide and confirmed it as a novel NIR probe for optical imaging of esophageal adenocarcinoma.

The in vitro testing validated the specificity of SNF-Cy5.5 to esophageal carcinoma cells (OE33 cells). in our previous study [11], SNF peptide selection was performed using phage display (Ph.D.-7, New England Biolabs, Beverly, MA) and a biopanning strategy based on esophageal carcinoma cells (OE33 cells). OE cells were well-validated cells models to investigate the specificity of SNF peptide to esophageal carcinoma cells in our several studies [11, 20]. Flow cytometry analysis showed SNF-Cy5.5 had a stronger binding to esophageal carcinoma (OE33 cells) than blocked SNF-Cy5.5. Besides, In line with the previous peptide concentration of 10 μM for cell staining [19], we used the same concentration peptide concentration in the current study. We demonstrated preferential binding to the plasma membrane of OE33 cells using confocal microscopy. The observation was similar to the other peptides which were found to bind to cell plasma membrane. For instance, Sturm et al. reported that the peptide ASYNYDA identified the cell surface target as Cyclophilin A (CypA). In the experiment of investigating the mechanism of the specific binding, we found the receptor of binding to SNF peptide was Epithelial cell adhesion molecule (EpCAM) [20]. EpCAM was observed on the cell surfaces of EAC [21], which is consistent to our observation under confocal microscopy.

The tumor targeting efficacy of SNF-Cy5.5 is also validated by in vivo optical imaging. In our current study, we built nude mice model bearing esophageal carcinoma cells (OE33 cells) tumors and validated the tumor targeting efficacy of SNF peptide to esophageal carcinoma cells. In vivo study showed a good uptake of such a low dose of our probe, which suggested a high binding property of SNF-Cy5.5. For the non-block group, the SNF-Cy5.5 presented a fast OE33 tumor targeting (0.5 h p.i.) and continuous high tumor-to-backgroud contrast during 24 h p.i, while, no probe intake was observed after 4 h p.i. for the non-block group, which revealed SNF-Cy5.5 is a target-specific probe. Furthermore, the fluorescence intensities of excised tumors were significantly higher in non-block group than those in blocked group, which is in line with the findings in vivo. It is worthwhile to notice that there is a relatively higher intake of SNF-Cy5.5 by the liver than other organs, including tumors and kidneys. Different short peptides conjugated with Cy5.5 were demonstrated with high intake by the liver [22, 23]. The higher uptake in the liver may be explained by the fact that the hepatic pathway is the main route of excretion.In addition, the kindey was also lighted up, which may suggest the renal pathway is the other important pathway of excretion.

Several tumor-targeted NIRF dyes were developed and investigated as promising probes for esophageal cancer. Chen jing et al. developed QRH*-KSP*-E3-Cy5.5 and showed 88% sensitivity and 87% specificity were found for the detection of either high-grade dysplasia (HGD) or EAC [24]. Lately, KSP-QRH-E3-IRDye800 was developed and the safety of orally administered KSP-QRH-E3-IRDye800 was evaluated for detection of esophageal neoplastic tissues in Phase 1A Study (Identifier: NCT03643068). In addition, Whitley MJ et al. developed LUM015, a novel PEGylated protease-activated far-red fluorescent imaging agent and found this probe could label tumor cells, and residual fluorescence within the tumor bed predicted local recurrence [25]. Currently, intravenously administered LUM015 was also evaluated for detect residual esophageal Cancer in clinical study (Identifier: NCT02584244). Both of the two probes were showed promising application prospects for detection of esophageal cancer. However, the rationales for detection and potential clinical application may be different. KSP-QRH-E3-IRDye800 were specific for human epithelial growth factor receptor 2 (HER2) and epidermal growth factor receptor (EGFR), which were have been found to be high-frequency gene amplified and overexpressed in early esophageal cancer [26, 27]. Thus, the probes of KSP-QRH-E3-IRDye800 and SNF-Cy5.5 were expected to be applied for early detection of early esophageal cancer under infra-red endoscopy. whereas, LUM015 were found to be bind to soft tissue sarcoma, thus, LUM015 was expected to identify microscopic residual disease within the tumor bed could be used to decrease the risk of a positive surgical margin, reduce the rate of re-resection, and tailor adjuvant therapy.

There are several limitations in our research. Firstly, we used an unlabeled SNF peptide as a control to investigate the specificity of SNF peptide to esophageal adenocarcinoma cells based on previous studies [23] and we found SNF-Cy5.5, rather than equal concentration of SNF-Cy5.5 with unlabeled SNF, bound specifically to esophageal adenocarcinoma in vitro and in vivo. However, Joshi et al. stated that development of a scrambled peptide was an alternative as a control [28], which inspired us and We will continue to investigate the scramble peptide in further work. Secondly, In addition, due to unproven safety of NIR fluorescent dye Cy5.5, the application of the dye is only limited to the imaging of animal models. indocyanine green (ICG) might be a better alternative. Compared with Cy5.5, ICG has longer wavelengths and established clinical applications [29]. Therefore, it is interesting to investigate SNF peptide labeled with ICG in the future research. Thirdly, though an excellent specific binding of SNF-Cy5.5 has been identified. However, the investigation of the effect of SNF peptide on tumor cell killing was not discussed in our study. So far, compared with specific antibodies with higher binding affinity, short peptides showed a stronger ability in tumor diagnosis application [28]. Short peptides in conjugation with nanoparticles seemed to be promising directions in further applications [30]. Therefore, it required further investigation of the value of SNF peptide on therapeutics at future. Fourthly, fluorescent histopathological staining in vivo study is important to better investigate sensitivity and specificity of the tracer. However, the fluorescent staining was not designed and included in the protocol of in vivo study. Therefore, further research should focus on fluorescent histopathological staining in vivo.

## Conclusion

Cy5.5-conjugated SNF was synthesized and characterized with a high efficiency and purity. The higher affinity, specificity, and tumor targeting efficacy of SNF-Cy5.5 were confirmed by in vitro and in vivo tests. SNF-Cy5.5 is a promising optical probe for the imaging of esophageal adenocarcinoma.

## Supplementary Information

**Additional file 1.** **Fig. 1**: The HPLC and MS results for SNF-Cy5.5.

## Data Availability

All data used to support the findings of this study are included within the article in Figs. 1, 2, 3 and 4 and supplementary material. All datasets on which the conclusions of the manuscript rely are presented in the paper.

## Abbreviations

NIR: Near-infrared
EAC: Esophageal adenocarcinoma
p.i: Post-injection
Cy5.5-SNF: SNF-Cy5.5-SNYMPL
HPLC: High Performance Liquid Chromatography
MS: Mass spectrum
IVIS: In vivo imaging system
FACS: Fluorescence-activated cell sorting
BEBM: Bronchial epithelial cell growth medium
DMSO: Dimethyl sulfoxide
DAPI: 4',6-Diamidino-2-phenylindole
TFA: Trifluoroacetic acid
ROI: Region-of-interest
SD: Standard deviation
FITC: Fluorescein isothiocyanate
