# Supplementary figures and images for: Targeted imaging of esophageal adenocarcinoma with a near-infrared fluorescent peptide
Source: BMC Gastroenterol. 2021 Jun 12;21:260. doi: 10.1186/s12876-021-01840-3 (PMC8199829; doi:10.1186/s12876-021-01840-3)

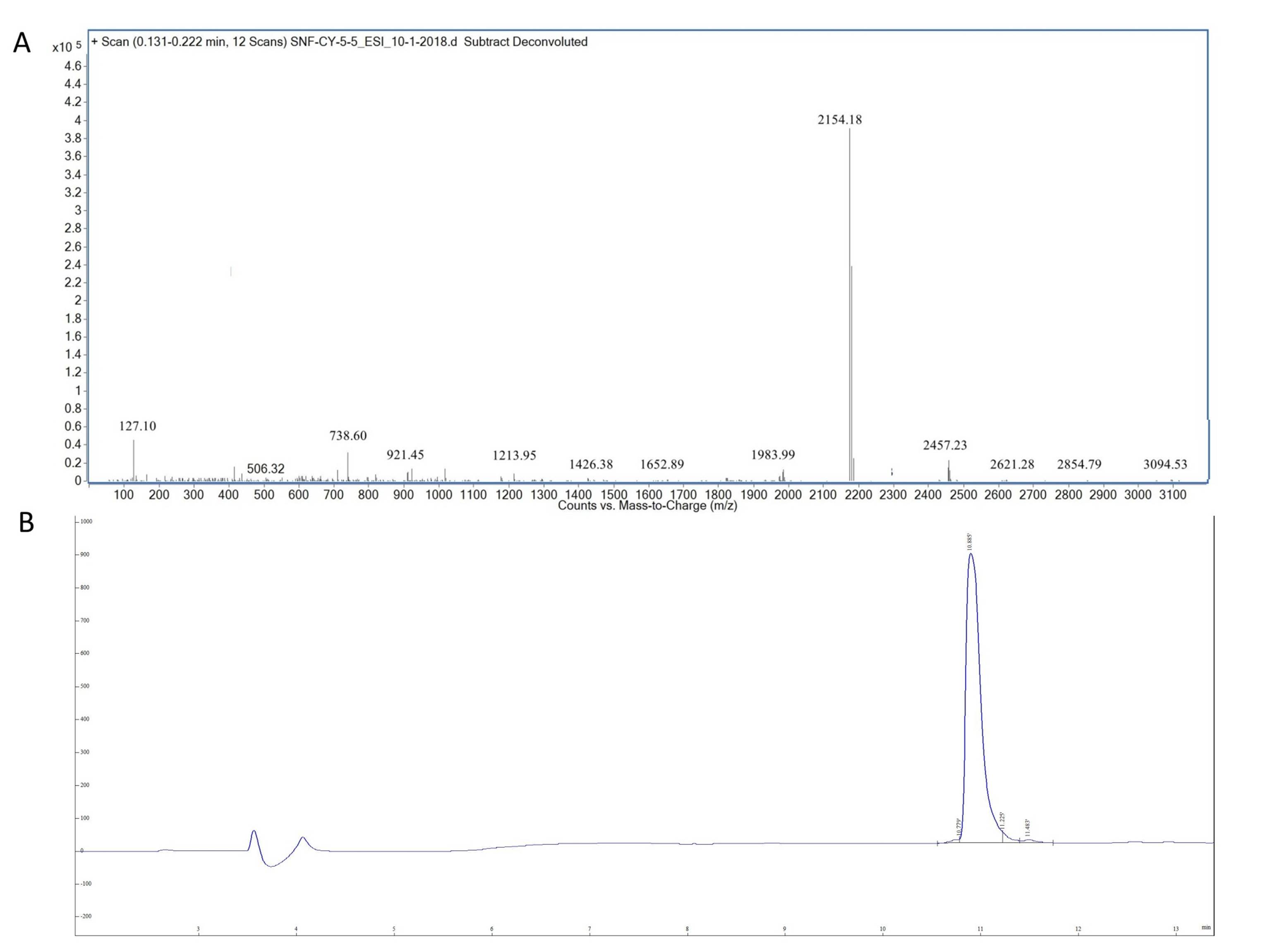

Supplement: Supplementary file 1 — Additional file 1. Fig. 1: The HPLC and MS results for SNF-Cy5.5. [file 12876_2021_1840_MOESM1_ESM.jpg]
